# The rising threat of climate change for arthropods from Earth's cold regions: Taxonomic rather than native status drives species sensitivity

**DOI:** 10.1111/gcb.16338

**Published:** 2022-07-22

**Authors:** David Renault, Camille Leclerc, Marc‐Antoine Colleu, Aude Boutet, Hoel Hotte, Hervé Colinet, Steven L. Chown, Peter Convey

**Affiliations:** ^1^ UMR 6553 Univ Rennes, CNRS, ECOBIO (Ecosystèmes, Biodiversité, Évolution) Rennes France; ^2^ INRAE, Aix‐Marseille Université, UMR RECOVER Aix‐en‐Provence France; ^3^ Nematology Unit, Plant Health Laboratory ANSES Le Rheu Cedex France; ^4^ Securing Antarctica's Environmental Future, School of Biological Sciences Monash University Melbourne Victoria Australia; ^5^ British Antarctic Survey, NERC Cambridge UK; ^6^ Department of Zoology University of Johannesburg Auckland Park South Africa

**Keywords:** arachnid, Araneae, Coleoptera, Diptera, heat exposure, insect, sub‐Antarctic islands, temperature, thermal fluctuations, warming

## Abstract

Polar and alpine regions are changing rapidly with global climate change. Yet, the impacts on biodiversity, especially on the invertebrate ectotherms which are dominant in these areas, remain poorly understood. Short‐term extreme temperature events, which are growing in frequency, are expected to have profound impacts on high‐latitude ectotherms, with native species being less resilient than their alien counterparts. Here, we examined in the laboratory the effects of short periodic exposures to thermal extremes on survival responses of seven native and two non‐native invertebrates from the sub‐Antarctic Islands. We found that survival of dipterans was significantly reduced under warming exposures, on average having median lethal times (LT_50_) of about 30 days in control conditions, which declined to about 20 days when exposed to daily short‐term maxima of 24°C. Conversely, coleopterans were either not, or were less, affected by the climatic scenarios applied, with predicted LT_50_ as high as 65 days under the warmest condition (daily exposures at 28°C for 2 h). The native spider *Myro kerguelensis* was characterized by an intermediate sensitivity when subjected to short‐term daily heat maxima. Our results unexpectedly revealed a taxonomic influence, with physiological sensitivity to heat differing between higher level taxa, but not between native and non‐native species representing the same higher taxon. The survival of a non‐native carabid beetle under the experimentally imposed conditions was very high, but similar to that of native beetles, while native and non‐native flies also exhibited very similar sensitivity to warming. As dipterans are a major element of diversity of sub‐Antarctic, Arctic and other cold ecosystems, such observations suggest that the increased occurrence of extreme, short‐term, thermal events could lead to large‐scale restructuring of key terrestrial ecosystem components both in ecosystems protected from and those exposed to the additional impacts of biological invasions.

## Graphical abstract

Effects of climatic changes on polar arthropods.

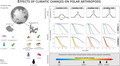

## 1 INTRODUCTION

Climate change is a major threat to biodiversity, among other consequences leading to phenological asynchrony, emerging infectious diseases and, ultimately, increasing extinction risks (Bellard et al., 2012; Iler et al., 2021; Lindgren et al., 2012; Maclean & Wilson, 2011; Renner & Zohner, 2016; Rödder et al., 2021; Román‐Palacios & Wiens, 2020). In parallel, multiple studies have reported climate‐induced range shifts of species to higher latitudes, altitudes or in other aspects of their overall geographical distribution (Chen et al., 2011; Spence & Tingley, 2020; Steinbauer et al., 2018; van Beest et al., 2021; Walther et al., 2002). Such displacement of species may carry high ecological costs, particularly in terms of impacts on pre‐existing native diversity. Where species are isolated (e.g. in island or island‐like ecosystems) or occupy discontinuous habitats (either naturally or as a result of anthropogenic fragmentation), long‐distance dispersal events between suitable areas may not be possible, in particular for organisms with limited dispersal capacities. As a result, the vulnerability of such organisms to already altered climates and predicted future change is a growing concern (Foden et al., 2019; Harter et al., 2015; Leclerc et al., 2020; Manes et al., 2021).

Ectotherms, whose biology is heavily influenced by physical environmental variation and its extremes, are likely to be among the most vulnerable to climate change (Burraco et al., 2020; Chou et al., 2019). Flexibility (phenotypic or genotypic) in a large range of ecophysiological traits is likely to be key in buffering the effects of changing environmental conditions and enabling organisms to maintain fitness. However, it has been suggested that this may not be sufficient to buffer the ever‐increasing impacts of warming on many ectothermic species (Gunderson & Stillman, 2015). In addition, while the amplitude of thermal variations and episodes of thermal extremes have been suggested to have the greatest influence on species range shifts (Germain & Lutz, 2020) and fitness (Colinet et al., 2015), a wide and increasing literature focuses on temperature means (Colinet et al., 2015).

Many studies have historically relied on standard protocols exposing organisms to biologically unrealistic rates of temperature change (Cannon, 1987; Worland & Convey, 2001). However, fewer investigations have assessed the effects of exposure to constant sub‐zero temperatures (Convey & Worland, 2000) as well as to temperature variability or repeated stress (Bale, 2002; Colinet et al., 2015) or the potentially synergistic consequences of exposure to multiple stresses (e.g. cross tolerance: Chown et al., 2007; Everatt et al., 2015). In the context of climate change, consequences will not simply be restricted to a consistent shift in the mean and range of temperature experienced, but also changes in the frequency, scale and rate of thermal variations experienced, as well as of exposure to extreme events (e.g. heat waves, heating events) (https://www.ipcc.ch/assessment‐report/ar6/; https://www.ipcc.ch/report/ar6/wg1/#FullReport; Jentsch & Beierkuhnlein, 2008; van de Pol et al., 2017). While these aspects have been studied for plants thriving in Arctic and alpine regions (e.g. the International Tundra Experiment ITEX, Henry & Molau, 1997; CREA Mont Blanc: https://creamontblanc.org/en/climate‐change‐and‐its‐impacts‐alps/; MIREN: mountaininvasions.org), fewer studies have addressed terrestrial invertebrates. Some studies have examined the responses of polar arthropods to warming (Bokhorst et al., 2012; Franken et al., 2018; Laparie & Renault, 2016), temperature extremes (Sørensen et al., 2019; Walzer et al., 2020) or increased thermal variability (Krog Noer et al., 2022; Lalouette et al., 2012; Sjursen et al., 2005) in the context of climate change. However, the effects of these aspects of increased thermal stress on the survival of arthropods from cold regions have been insufficiently examined to date, as highlighted by Bahrndorff et al. (2021). These authors pointed out the limited information available to aid our understanding of the capacity of terrestrial arthropods from polar regions to respond to future temperature warming, thus preventing the development of accurate predictions of the resistance/resilience and future distributions of such species in the context of climate change. This knowledge is additionally critical for the implementation of conservation strategies, and establishment of roadmaps for biodiversity conservation (Wilson & Fox, 2021).

Climatic conditions experienced in cold environments globally (polar and alpine regions, tundra) have been changing more rapidly than in other parts of the world in recent decades. In parts of the polar regions, particularly with reference to warming, this phenomenon is referred to as polar amplification (ACIA, 2005; Pedersen et al., 2022; Turner & Overland, 2009). A number of significant consequences of these changes have already been reported or predicted. They include (a) retreat of glaciers and loss of floating ice shelves (Cook et al., 2005; Turner & Overland, 2009), (b) reduction in the number of freezing days during winter periods (Lebouvier et al., 2011), winter thaw events and changes in timing of spring thaw and winter freeze (Pedersen et al., 2022; Rixen et al., 2022), (c) reduced precipitation and more frequent summer drought events (McClelland et al., 2018), (d) altered plant communities (Chapuis et al., 2004; Collins et al., 2022; Myers‐Smith, Grabowski, et al., 2019; Myers‐Smith, Thomas, et al., 2019; Prevéy et al., 2019), (e) increased probabilities of establishment and potential distributions of non‐native plants and arthropods (Chown et al., 2012; Convey & Peck, 2019; Pertierra et al., 2017, 2020), and (f) distribution expansion in non‐native species already established (Bartlett et al., 2020; Le Roux et al., 2013; Lebouvier et al., 2020) and of native species (Contador et al., 2020; Duffy et al., 2017).

A number of studies have reported data on the global effects of climate change on arthropods from cold environments at the population and community levels (Bonelli et al., 2022; Chown & Brooks, 2019). However, the responses of polar (Arctic, sub‐Antarctic, Antarctic) ectothermic species to current and future environmental variability, stochastic environmental fluctuations, or extreme events are rarely addressed and thus remain under‐represented in the literature (Bahrndorff et al., 2021; Høye, 2020; but see a range of experimental environmental manipulation studies, as reviewed by Bokhorst et al., 2013). Increased understanding of the impacts of environmental change, particularly relating to temperature, on the life cycles of these under‐examined taxa is of particular importance for understanding of their ecological function and potential changes to this. It is also required in evolutionary or biogeographical studies comparing between regions, such as the sub‐Antarctic islands, characterized by different climatic conditions (Leihy et al., 2018). The sub‐Antarctic zone includes several distinct ecoregions, which differ in terms of landscapes, plant and invertebrate communities, and meteorological conditions (Leihy et al., 2018). In the sub‐Antarctic, arthropods have evolved under chronically low but relatively stable environmental conditions (in terms of limited annual thermal variation and high relative humidity; Convey, 1996a, 1996b). They are thus expected to exhibit stenotypic characteristics, and a level of thermal adaptation to their native ecoregion, whereas non‐native incoming species, typically originating from temperate (often boreal) regions are likely to exhibit more generalist eurythermal characteristics (Barendse & Chown, 2000; Chown & Convey, 2016; Phillips et al., 2020; Slabber et al., 2007).

This study set out to determine if the level of physiological tolerance of sub‐Antarctic arthropods is sufficient to cope with the challenges posed by their rapidly changing environments, and in particular by extreme events which are increasingly occurring in the region (Feron et al., 2021; Robinson et al., 2020; Turner et al., 2021). Increases in the frequency, severity and duration of exposure to temperature extremes are anticipated in the near future, and increased temperature variation poses a greater risk to species than does mean temperature change (Vasseur et al., 2014).

The sub‐Antarctic Kerguelen and Crozet archipelagos are located in the Indian Ocean sector of the Southern Ocean. These islands are also characterized by the highly depauperate macro‐arthropod fauna they host (Hullé & Vernon, 2021), with for instance nine native spider species, 31 native beetle species (of which 22 are weevils) including 24 species endemic to the region, and 27 native fly species of which 10 are endemic to the region (Hullé & Vernon, 2021). Of note, there are far fewer endemic terrestrial arthropod species at the Kerguelen Islands as compared with Possession Island (Crozet archipelago), with the Kerguelen Islands hosting for instance only six dipteran species native to the Southern Indian Ocean or from the sub‐Antarctic region (Hullé & Vernon, 2021). Their position near the boundary between the oceanic polar and subtropical fronts results in them having relatively stable, yet distinct, cold oceanic climates with mean annual temperatures of ~5°C and mean monthly surface temperatures ranging between +1.3 and 9.0°C (Lebouvier et al., 2011). Over recent decades, these archipelagoes have experienced increases in mean air temperature. In the Crozet archipelago, the increase in mean annual temperature has been approximately 0.5°C since the 1980s (source: Météo France, https://donneespubliques.meteofrance.fr/). There has been a more marked increase in the Kerguelen archipelago, amounting to 1.3°C since the 1960s (Lebouvier et al., 2011). Additionally, multiple extreme warm events have occurred during the austral summers of the second half of the 1990s, for example with air temperature briefly reaching up to 23°C (source: Météo France, https://donneespubliques.meteofrance.fr/).

To examine the relative importance of taxonomy (beetle, fly, spider), biogeographical origin (Crozet or Kerguelen Islands) and native vs. non‐native status on arthropod thermal sensitivity, a proportion of the native species from the Crozet and Kerguelen archipelagoes, and some non‐native counterparts, were experimentally exposed to different scenarios of climate warming in the laboratory. One of these simulated the contemporary summer climate of the Crozet and Kerguelen Islands (temperature oscillating between 4 and 12°C), and three other scenarios simulated small (4–20°C), moderate (4–24°C) and large (4–28°C) changes in the thermal ranges that might be experienced in future. Through these experiments, we aimed to provide a robust assessment of the abilities of these sub‐Antarctic arthropods to respond to plausible and ecologically relevant environmental changes.

## 2 MATERIALS AND METHODS

### 2.1 Arthropod sampling

Adults of nine arthropod species (Table 1) were collected in February–March (austral summer) 2014, 2015 and 2016 at the Crozet (46°25′S; 51°51′E) and Kerguelen (48°25′–50S; 68°27′–70°35′E) Islands. One species, the native fly *Anatalanta aptera* was sampled from both archipelagos. Three native species (the carabid beetle *Amblystogenium pacificum*, the fly *Anatalanta crozetensis* and the spider *Myro kerguelenensis*) were sampled at the Crozet archipelago alone, and five species (the native flies *Amalopteryx maritima* and *Calycopteryx moseleyi*, the native weevil *Bothrometopus brevis*, the non‐native fly *Fucellia tergina* [a common intertidal fly in Pacific and Atlantic regions] and the non‐native carabid *Merizodus soledadinus* [an insect of austral origin, distributed in Patagonia and Falkland Islands]) were sampled at the Kerguelen Islands alone. For *C. moseleyi*, two distinct ecotypes were sampled, one from the foreshore and the other from Kerguelen cabbage plants (*Pringlea antiscorbutica*, Brassicaceae). Sub‐Antarctic Islands are generally characterized by low species diversity in comparison with other eco‐regions of the world. This is particularly true for the Kerguelen Islands, which host six dipterans native from the Southern Indian Ocean or from the sub‐Antarctic regions, meaning that our study included 50% of the fly diversity from these islands (Hullé & Vernon, 2021). Many species additionally occur at low densities, and some are rare. Of necessity, sampling and investigation had to be restricted to those species present at sufficient density (i.e. which could be collected without causing significant impact on their local populations), and to species whose adult stage duration was at least 20–30 days to complete our experimental protocols. Also, to limit sampling impacts on these UNESCO World Heritage listed archipelagoes, a varying number of individuals were sampled each year over the three consecutive years of the study (Supplementary Material S1). For some of the most stressful treatments (‘4–24’ and ‘4–28’, see below for treatment descriptions), the survival experiments had to be re‐run with different individuals to ensure that observation times allowed us to generate reliable survival curves (total numbers of tested individuals are given in Supplementary Material S1). These duplicates of the survival experiments also confirmed the consistency of the results obtained.

**TABLE 1:** Arthropod species from the Kerguelen and Crozet archipelagoes used for the assessment of the effects of different warming scenarios (condition tested, see Section 2 for details) on survival

Species	Order	Biogeographic origin	Native/non‐native status	Condition tested (see Section 2 for details)			
				‘4–12’	‘4–20’	‘4–24’	‘4–28’
*Anatalanta crozetensis*	Diptera	Crozet	Native	X	X	X	X
*Anatalanta aptera*	Diptera	Crozet; Kerguelen	Native	X	X	X	X
*Amalopteryx maritima*	Diptera	Kerguelen	Native	X	X	X	X
*Calycopteryx moseleyi*	Diptera	Kerguelen	Native	X	X	X	X
*Fucellia tergina*	Diptera	Kerguelen	Non‐native	‐	‐	X	X
*Amblystogenium pacificum*	Coleoptera	Crozet	Native	X	X	X	X
*Bothrometopus brevis*	Coleoptera	Kerguelen	Native	‐	‐	X	X
*Merizodus soledadinus*	Coleoptera	Kerguelen	Non‐native	X	X	X	X
*Myro kerguelenensis*	Araneae	Crozet	Native	X	X	X	X

After collection, individuals were placed into plastic boxes containing substrate from the sampling sites, *Pringlea* leaves or seaweed (for species sampled from littoral habitats) and stored under controlled conditions (5–6°C) for 2 days prior to survival experiments commencing. Based on the metabolic phenotypes of a range of insects (Colinet & Renault, 2012; Enriquez et al., 2018), this duration is considered sufficient (Engell Dahl et al., 2019) for smoothing possible temperature and transport differences experienced by the different tested arthropods. A single species was maintained in each plastic box. For each species, groups of 10 individuals were then transferred to Petri dishes (40 Petri dishes per species, or per ecotype), where they were not provided with food but were supplied with water ad libitum.

### 2.2 Survival of adult arthropods experimentally exposed to increased temperatures

**FIGURE 1:**
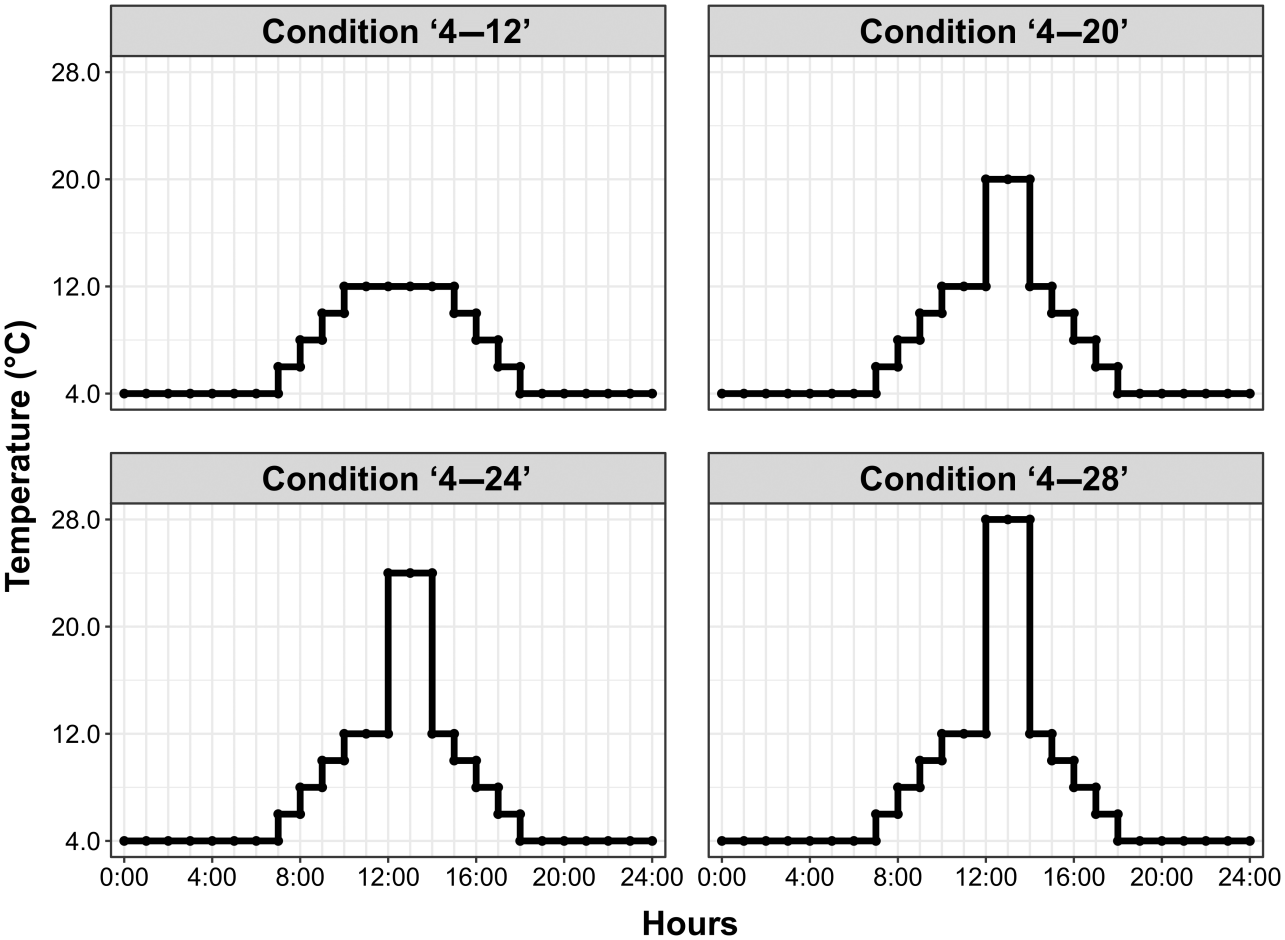
The four daily experimental thermal fluctuations. Condition ‘4–12’: from 4 to 12°C; Condition ‘4–20’: from 4 to 20°C; Condition ‘4–24’: from 4 to 24°C; Condition ‘4–28’: from 4 to 28°C.

The various different models considered by the Intergovernmental Panel on Climate Change in its sixth assessment report predict that global mean air temperature could rise by between 1.5 and 4.0°C by the end of the 21st century (https://www.ipcc.ch/assessment‐report/ar6/, https://www.ipcc.ch/report/ar6/wg1/#FullReport). Based on the scenarios generated from these models, four experimental thermal regimes were used to assess the responses of native and non‐native sub‐Antarctic arthropods to warming: ‘4–12’ (daily temperature peak at 12°C for 5 h) was designed to mimic the thermal variability currently experienced over the typical daily cycle during summer (see also Leihy et al. (2018) for ground‐truth climatic information from long‐term monitoring in subsurface situations), this temperature regime is thus regarded as the control; ‘4–20’ (daily temperature increasing to 12°C, and further peaking at 20°C for 2 h) represents a scenario of mild heating event; ‘4–24’ (daily temperature increasing to 12°C, and further peaking at 24°C for 2 h) represents a moderate heating event; ‘4–28’ (daily temperature increasing to 12°C, and further peaking at 28°C for 2 h) represents a severe heating event (Figure 1). For the warmer conditions (‘4–24’ and ‘4–28’), effective daily changes in temperature and relative humidity values are presented in Supplementary Material S2. For each temperature condition and species or ecotype, 10 Petri dishes (each with 10 individuals) were placed into incubators (Panasonic MIR 154). All experiments started in the evening so that the animals were exposed to 4°C for at least 12 h overnight in all thermal regimes before the daily temperature increase commenced. In all experiments, the day:night cycle duration was 14:10 h. Of note, initial examination of the data obtained suggested that the taxonomic identity could be as important as the native or non‐native status of the species, with similar survival patterns seen in the carabid beetles *A. pacificum* and *M. soledadinus*. To better examine this aspect, the non‐native fly *Fucellia tergina* and the native beetle *B. brevis* were added to the study in the third year of the experiment, and their responses to conditions ‘4–24’ and ‘4–28’ were assessed.

After 5 days of exposure to one of the four regimes (4–12, 4–20, 4–24 or 4–28), one Petri dish (i.e. 10 individuals) per experimental condition was returned to a constant temperature of 7–8°C for 24 h. After this 24 h of recovery period (preliminary tests revealed that survival remained similar from 24 h onwards), survival was assessed as follows: ‘alive’, where individuals showed normal locomotory activities, ‘sublethal effects’, where only movements of appendages were visible, or ‘lethal effects’, where no evidence of movement was observed even after stimulation with a paintbrush. Subsequently, the remaining Petri dishes were removed individually at regular intervals (every ca. 2–5 days, depending on the experimental condition and species tested) and survival was assessed as described above. All experiments were stopped after 39 days, or earlier in experimental situations that resulted in the mortality of all individuals.

### 2.3 Statistical analyses

As some species' responses to experimental thermal regimes were assessed twice in different years (see Supplementary Material S1), a log‐rank test was applied to compare species survival curves between years using the *survfit()* function from the R package *survival* (Therneau, 2021). No differences were detected between sampling years for any species' responses to experimental thermal regimes. Thus, data from all years were pooled in the subsequent analyses. Second, as survival curves can only be fitted with binary encoded data (i.e. dead: 1, alive: 0), the three categories (alive, unfit or dead individuals) were reduced to two. From an ecological point of view, we tested whether individuals suffering sublethal effects could be combined with those lethally affected, as the former appeared not to recover owing to the amount of damage accumulated, and thus could not contribute to population dynamics (through mating, oviposition, etc.). As previously, a log‐rank test was applied, this time to compare species survival curves including unfit individuals as dead or not. No significant difference was found, and individuals suffering sublethal effects were therefore considered as dead for the purpose of this study.

After this two‐step check of the dataset, survival curves were fitted using a parametric accelerated failure model assuming a Weibull distribution by applying the *survreg()* function from the R package *survival* (Therneau, 2021). Among the available accelerated failure models, the Weibull distribution was used as it produced the minimum error deviance. To assess the effect of the different warming scenarios on the survival of each species, and to compare survival across species under each scenario, pairwise comparisons using the Bonferroni correction were applied using the *pairwise_survdiff()* function from the R package *survminer* (Kassambara et al., 2021). Furthermore, the median lethal times (LT_50_) of species exposed to the warming conditions were extracted from the fitted survival curves. LT_50_ values were log‐transformed to correct inherent skewness before being mean centred and expressed in units of standard deviation. To investigate the influence of warming conditions, biogeographical origin, taxonomic order and native/non‐native status on LT_50_, we applied a Bayesian phylogenetic mixed model from the R package *MCMCglmm* (Hadfield, 2010) to account for non‐independence among closely related species and multiple data points per species. This approach included the phylogenetic relationships among species as a random effect to correct for the shared ancestry. To estimate phylogenetic relatedness, a phylogeny that only included the nine species was extracted from the Open Tree of Life using the R package *rotl* (Michonneau et al., 2016). We used an uninformative prior (with variance set to 1 and belief parameter set to 0.002) for both fixed and random effects (Hadfield, 2010) and ran the model for 100,000 iterations with a thinning value of 10 after a burn‐in of 250. We checked model convergence using the Gelman–Rubin statistic (Gelman & Rubin, 1992). Potential scale reduction values were all less than 1.1 and the autocorrelations of posterior probabilities were all less than 0.2. For species with missing LT_50_ values, MCMCglmm samples them under the assumption of missing at random process. All analyses were conducted with R version 4.1.0 (R Development Core Team, 2015).

## 3 RESULTS

### 3.1 Effect of thermally stressful conditions

**FIGURE 2:**
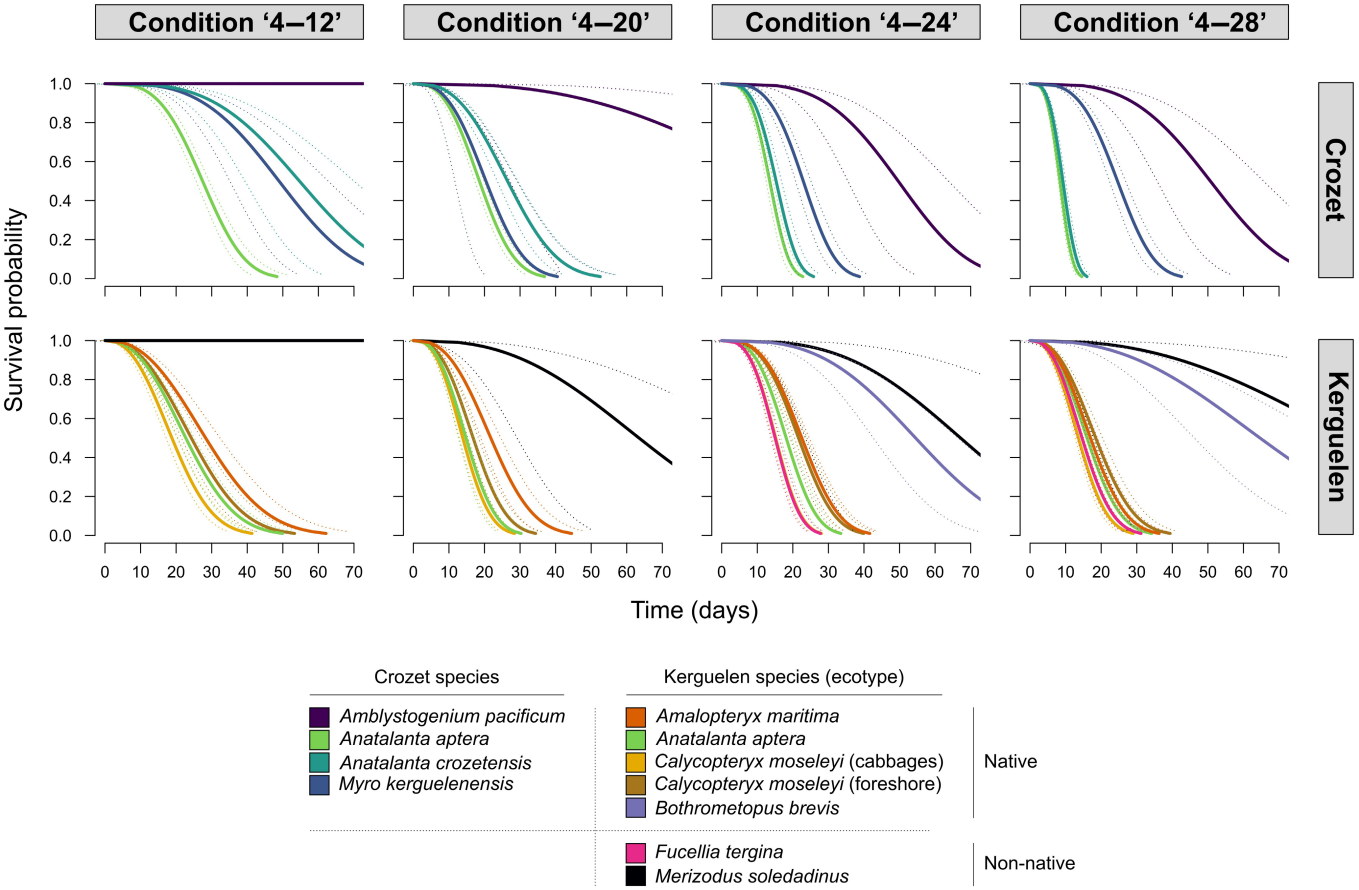
Fitted survival curves (Weibull distribution) of native and non‐native arthropods from the Kerguelen and Crozet Islands under different warming scenarios. Solid lines represent predicted values of survival probability and associated dotted lines represent 95% confidence interval. Condition ‘4–12’: from 4 to 12°C; Condition ‘4–20’: from 4 to 20°C; Condition ‘4–24’: from 4 to 24°C; Condition ‘4–28’: from 4 to 28°C.

Survival patterns of the tested species were significantly influenced by the thermal regimes (Figure 2; Table 2). Exposure to repeated mild (‘4–20’, daily short‐term maximum of 20°C), moderate (‘4–24’, daily short‐term maximum of 24°C) or severe (‘4–28’, daily short‐term maximum of 28°C) warming events led to progressively lower species survivorship as the daily temperature peak increased, in comparison to the survivorship obtained in the ‘4–12’ control condition (Table 2). As an illustrative example, the survivorship of *A. crozetensis*, whose individuals had a median lethal time (LT_50_) of 54 days in the condition ‘4–12’, was highly significantly reduced to 9 days in the condition ‘4–28’ (Table 3).

**TABLE 2:**
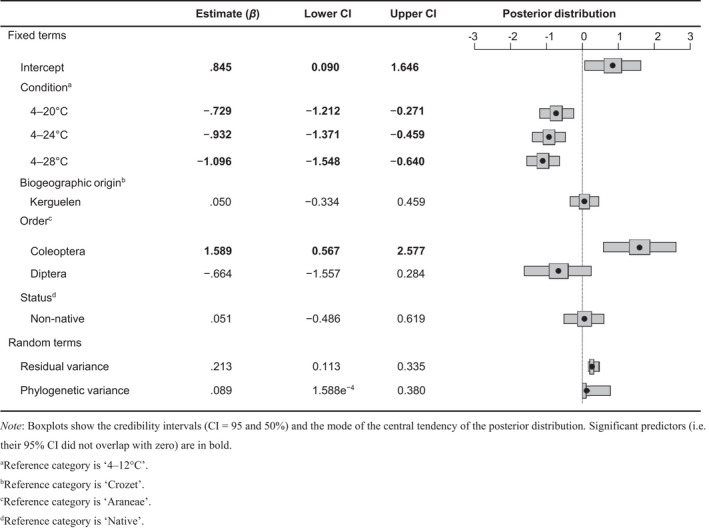
Model explaining the time to 50% species survival (LT_50_, in days) by the effect of warming condition, biogeographical origin (i.e. Crozet/Kerguelen islands), taxonomic order (Order) and native/non‐native status of species (Status)

**TABLE 3:** Time to 50% mortality (LT_50_, in days) of the different tested species subjected to four experimentally applied warming scenarios

	Lethal time 50% (±95% CI)			
Species	4–12°C	4–20°C	4–24°C	4–28°C
Diptera				
*Anatalanta crozetensis* (NS, Crozet)	54.6 (±15.1)	26.4 (±2.8)	15.2 (±1.4)	9.2 (±0.7)
*Anatalanta aptera* (NS, Crozet)	27.4 (±1.8)	18.5 (±1.2)	13.4 (±0.9)	8.3 (±0.4)
*Anatalanta aptera* (NS, Kerguelen)	22.7 (±2.3)	14.8 (±2.1)	18.1 (±1.4)	16.0 (±1.3)
*Amalopteryx maritima* (NS, Kerguelen)	28.2 (±3.9)	21.7 (±2.7)	22.5 (±2.0)	16.9 (±1.9)
*Calycopteryx moseleyi* (NS, Kerguelen)^a^	24.2 (±2.7)	16.8 (±1.4)	21.6 (±2.3)	18.4 (±1.9)
*Calycopteryx moseleyi* (NS, Kerguelen)^b^	18.8 (±1.6)	13.8 (±0.9)	15.1 (±0.9)	13.5 (±0.9)
*Fucellia tergina* (NNS, Kerguelen)	NA	NA	15.0 (±1.1)	14.6 (±1.3)
Coleoptera				
*Amblystogenium pacificum* (NS, Crozet)	+∞	103.3 (±75.3)	49.6 (±14.0)	50.6 (±14.8)
*Bothrometopus brevis* (NS, Kerguelen)	NA	NA	54.5 (±11.7)	64.6 (±18.2)
*Merizodus soledadinus* (NNS, Kerguelen)	+∞	63.4 (±33.9)	67.1 (±43.1)	89.6 (±71.0)
Araneae				
*Myro kerguelenensis* (NS, Crozet)	48.9 (±13.6)	20.4 (±8.6)	22.9 (±2.1)	24.5 (±2.2)

*Note*: It was not possible to compute LT_50_ for *A. pacificum* and *M. soledadinus* under the condition ‘4–12°C’ as no mortality was recorded.

Abbreviations: CI, confidence interval; NNS, non‐native species; NS, native species.

^a^ Foreshore ecotype.

^b^ Kerguelen cabbage ecotype.

### 3.2 Effect of native/non‐native status

Survival patterns were overall not influenced by the status of the species (native, non‐native), with survivorship under the four tested thermal conditions being similar in native and non‐native taxa (Table 2; in comparison to the survivorship measured in native arthropods, the statistics obtained for the non‐natives were: *β* = .051, 95% confidence interval [CI] = −0.486 to 0.619). The native weevil *B. brevis* was characterized by a relatively good ability to tolerate moderate (‘4–24’) and high (‘4–28’) warming scenarios, and the survival of *B. brevis* did not differ significantly from that of the non‐native and native carabid beetles *M. soledadinus* and *A. pacificum* (Figure 2; Supplementary Material S3). The non‐native fly species *F. tergina* exhibited the lowest survival duration under ‘4–24’ and showed similar survival to that of the native flies sampled from the Kerguelen Islands under ‘4–28’, with the survival curves of the native and non‐native flies grouping together under this harshest regime (Figure 2; Supplementary Material S3).

### 3.3 Effect of taxonomic order

Survival patterns were significantly influenced by the taxonomic identity of the species (particularly at the order level). Coleoptera were characterized by the highest survivorship across all the assayed warming scenarios. Then, Araneae and Diptera had a similar sensitivity to warming, although Diptera tended to be the most susceptible to the warming scenarios tested (Table 2).

The survival of *M. kerguelensis* (Araneae, Crozet Islands) reduced as soon as warming occurred, even in the mild short‐term heat exposure (‘4–20’); however mortality, and thus the effects of warming, did not further increase when the spiders were exposed to harsher temperature conditions (‘4–24’ and ‘4–28’). As a result, even though the survival of *M. kerguelensis* was similar to that of dipterans under ‘4–20’, their survival was significantly greater than that of dipterans, although still lower than coleopterans, under the warmer conditions (‘4–24’ and ‘4–28’) (Figure 2; Supplementary Materials S4 and S5). Coleoptera were the only group for which no mortality was recorded under the control condition (‘4–12’, Figure 2; Supplementary Material S4).

### 3.4 Effect of biogeographical origin

Survival patterns were not influenced by the biogeographical origin of the tested arthropods. Survivorship under thermal conditions did not differ significantly between the taxa collected at the Kerguelen and Crozet archipelagoes (Table 2; in comparison to the survivorship of taxa collected from the Crozet Islands, the statistics for taxa collected from the Kerguelen Islands were: *β* = .050, 95% CI = −0.334 to 0.459). Although survival of the native dipteran *A. aptera* from the Crozet Islands was more strongly reduced under the harshest warming scenario (‘4–28’) as compared with dipterans collected from the Kerguelen Islands (Figures 2; [Link], [Link]), the survivorship of *A. aptera* from both Crozet and Kerguelen Islands was similar under ‘4–20’ (*p* = .186, Supplementary Material S5).

## 4 DISCUSSION

Native arthropods of the sub‐Antarctic islands have evolved under chronically cool thermal conditions with limited annual thermal variability (Convey, 1996a, 1996b; Peck et al., 2006), although also potentially facing periodically extreme winter events if not protected by snow or vegetation cover. In recent decades, climate has changed significantly in these regions, for example with winters becoming warmer and summers drier (Lebouvier et al., 2011; McClelland et al., 2018). Globally, general warming trends are accompanied by alterations in the pattern and scale of diel variations, increasing the likelihood of occurrence of exceptional warm events during the summer. In the context of ongoing climatic changes taking place in many cold environments worldwide (Antarctic, Arctic, alpine, Tibetan plateau), we attempted to assess whether the levels of basal thermal tolerance of a selection of both native and non‐native arthropods present on the Crozet and Kerguelen archipelagos are sufficient to give them resilience to the rapidly changing climates.

Climatic characteristics, including mean temperatures, diel and seasonal temperature variation and extreme temperature events, are strong drivers of thermal limits and tolerance ranges in insects (Clusella‐Trullas et al., 2011), as defined in the climatic variability hypothesis (Stevens, 1989). The often endemic insects native to the sub‐Antarctic islands have evolved in these thermally buffered environments and might, therefore, be hypothesized to have limited scope in thermal tolerance and plasticity in comparison to non‐native species, of which many are of temperate origin. Our data demonstrate that exposure to different patterns of thermal variance, in line with the predictions of different climate scenarios, had a strong effect on the survival of the sub‐Antarctic insects tested. A similar conclusion was drawn by Bozinovic et al. (2016) in a study of the fruit fly *Drosophila melanogaster*. In our study, the daily exposure of the insects for 2 h at 24°C, a temperature well below the critical thermal maxima of native sub‐Antarctic species (which are in the range 32–35°C; Jumbam et al., 2008; Klok & Chown, 2001), strongly reduced their survival relative to control conditions, in particular that of dipterans native to the Crozet Islands. Even the lowest warming simulation resulted in a significant decrease in dipteran survival. The phenotypic plasticity of insects is often more limited at high than low temperatures (Addo‐Bediako et al., 2000; Hoffmann et al., 2013; Lalouette et al., 2012), suggesting that little or no increase in survival through acclimation was likely to have occurred over the course of experiments such as described here.

The impact of diel thermal variability on the survival of sub‐Antarctic insects also questions the validity of thermal safety margins, that is, the difference between body temperature and temperature thresholds for function or lethality (Gunderson & Leal, 2015), as a means of estimating vulnerability. Studies have predicted that ectotherms, including insects, from higher southern or northern latitudes should be less sensitive to warming as compared with lower latitude species which already thrive in climatic conditions close to their optimal temperature (Deutsch et al., 2008). Different thermal vulnerability indices can be measured for obtaining estimates of the sensitivity of species to warming (Clusella‐Trullas et al., 2021): some of these investigations rely on critical thermal limits, while others are based on survival responses to lethal or sublethal stress exposures, as used in the present study. Critical thermal maxima correspond to the ultimate temperature an insect can survive and, thus, only brief heat exposures can be applied which may be less relevant for predictions of the effects of climate warming. As a result, sub‐critical thermal limits, which occur at lower temperatures and can be experienced from minutes to hours (Chown & Klok, 1997), are more ecologically relevant and recommended (Braschler et al., 2021).

Here, when the dipterans *A. crozetensis* and *A. aptera* were exposed to 24°C for 2 h daily, their LT_50_s were significantly reduced (Table 3). Even if these findings are consistent with the studies of Nyamukondiwa et al. (2018) and Terblanche et al. (2010), who reported reduced tolerance to high temperatures when thermal variability was increased, we have little knowledge of the sublethal effects of short‐term exposure to moderately or severely stressful temperatures in arthropods from cold environments. Daily temperature extremes, as distinct from mean temperature, can strongly affect the performance of insects by depressing fecundity, longevity and, ultimately, survival (Ma et al., 2015). Recently, it has been demonstrated that short‐term heating events, in the form of 4 h exposures of drosophilids to 38°C, can permanently sterilize males (Walsh et al., 2021). Moreover, fertility loss generally occurs at temperatures lower than those recorded for upper thermal limits, and better predicts the geographical distribution of the species (Parratt et al., 2021). If similar patterns occur in sub‐Antarctic dipterans, the sensitivity of individuals to increased temperature may be even higher than identified in this study, and the consequences for population dynamics alarming.

Most of the tested species are flightless (only the non‐native fly *F. tergina* has the ability to fly), and some of them share the same terrestrial habitats, thus experiencing similar soil surface temperatures. In our experimental design, any behavioural responses to thermal stress were not considered, that is, no heat escape response or selection of sheltered habitats was possible. Behavioural responses can be important in arthropods facing thermal stresses (Ma & Ma, 2012; Malmos et al., 2021), and further studies should compare the heat escape responses of the species. Such behavioural responses could lower the importance of physiological responses in the survival of heat exposures (Bogert, 1949) and, from our findings, could play a critical role for flies that can select microclimates (see Pincebourde & Woods, 2020 for discussion of the importance of microclimates) in the field during heating events.

The native coleopterans *A. pacificum* and *B. brevis* were either not, or were less, affected by the climatic scenarios applied in this study than were the dipterans. This suggests a taxonomic influence, with physiological sensitivity to heat systematically differing between higher level taxa. Under exposure to varying thermal conditions, insects may recover from heat injuries during the cooler part of the temperature regime (Colinet et al., 2015; Lalouette et al., 2011; Renault et al., 2004). Klepsatel et al. (2016) suggested that heat stress increases the cost of somatic maintenance, as revealed by the progressive depletion of triglyceride reserves in *D. melanogaster*. However, the differing responses of dipterans and coleopterans remain hard to explain, as it is unlikely that the cost of somatic maintenance is close to null in the latter. Energy loss could have been greater in flies, which would thus require higher food intake when daily heat maxima become more extreme. However, survival of the flies was significantly impaired as soon as the temperature was warmed which, rather, suggests that the occurrence of deleterious effects of heat is most probably progressive, starting from macromolecules, and then extending to cells, tissues and organs (Denlinger & Yocum, 1998). The cuticle of insects is very sensitive to thermal fluctuations and its wax layer can be altered, in turn increasing the rates of desiccation. Moreover, our monitoring of the ambient conditions inside the Petri dishes showed that warming was accompanied with decreased relative humidity, in particular at ‘4–28’ (Supplementary Material S2). We thus suggest that differences in cuticular permeability between dipterans and coleopterans may underlie the striking differences in their vulnerability to heat exposure and accompanying changes in relative humidity conditions. The results from a desiccation resistance experiment conducted by Ring et al. (1990) support this idea, with the ability of coleopterans to handle low humidity conditions being better than that of spiders and significantly better than flies. However, these authors only considered four sub‐Antarctic species, thus limiting the ability to draw a robust conclusion.

At the Kerguelen Islands, the aggressively carnivorous ground beetle *M. soledadinus* is the only invasive insect originating from the southern cold temperate region, where it is native to Patagonia, Tierra del Fuego and the Falkland Islands (Lebouvier et al., 2020). Laparie and Renault (2016) assessed the metabolic phenotypes of adult *M. soledadinus* kept at different temperatures for 2‐week periods and did not find evidence for metabotypes that would characterize exposure to acute stress in insects constantly exposed at 20°C. Studies of this species have consistently concluded that the thermal tolerance of *M. soledadinus* exceeds the temperature range and thermal variability currently experienced in its distribution on the Kerguelen Islands. Our findings are consistent with these investigations and provide further confirmation that future climate warming will therefore accelerate the progressive colonization of the archipelago by this beetle (Engell Dahl et al., 2019; Ouisse et al., 2020).

Cold environments have experienced multiple incursions by non‐native species (i.e. invasion meltdown). Often establishment, and subsequent changes in the distribution of the non‐native species, have been suggested to be facilitated by climate change. Consistent with this, Phillips et al. (2020) reported that non‐native collembolans were characterized by significantly higher critical thermal maxima than their native counterparts, although thermal plasticity differed little between them. Similar findings were reported by Janion‐Scheepers et al. (2018) with warming tolerance being higher in non‐native springtails over the climatic gradient they studied. In the context of climate change, we hypothesized that non‐native species thriving in warm environments in their native range should be characterized by better abilities to deal with thermal extremes in the invaded range, that is, characteristics of climatic niches would be predictors of insect abilities to cope with warming. Thus, warming should be less detrimental to non‐native arthropods as compared with native species, in particular because the latter are often close their climatic limits (Norberg et al., 2012). A notable conclusion from our study is the strength of influence that the higher taxon has on the sensitivity to warming independent of the native/non‐native status of the arthropods. The survival of the non‐native carabid beetle under the experimentally imposed conditions was very high, but was similar to that of the native beetle *A. pacificum*. In an analogous fashion, the exposure survival of the non‐native fly *F. tergina* reduced very rapidly when exposed to daily heat maxima, as also observed in native flies.

Our multi‐species investigations provide in‐depth and novel understanding of the effects of climatic changes on the survival of ground‐dwelling arthropods of cold environments whose sensitivity to future climate change has been insufficiently studied to date (Bahrndorff et al., 2021; Franken et al., 2018; Høye, 2020) as compared with plants. Of note, the arthropods studied have been repeatedly exposed to short‐term maxima, while extreme warming events may occur more episodically in the field. We acknowledge that the repetition of stress may have been deleterious to the arthropods (Marshall & Sinclair, 2012), which might have led to overestimation of the warming impacts on the survival durations. However, the same experimental design was used for all species, thus allowing to underscore their different abilities to handle exposure to short‐term thermal maxima. Our results also confirm that exposure to sub‐critical temperatures can more appropriately capture the effects of warming on ectotherms as compared with the use of more stressful critical thermal limits (Braschler et al., 2021). Our data provide the first evidence that dipterans may be much more at risk in cold regions subject to warming. A plausible underlying reason for this might be the different cuticle properties characterizing dipterans in comparison with coleopterans. Our study highlights the risks facing terrestrial arthropods from cold regions of being negatively impacted by climate change in the near future.

## 5 CONCLUSIONS

The Earth's cold regions—Antarctic, Arctic, alpine, Tibetan plateau—are particularly exposed to the consequences of global climate change, with warming often taking place at rates amplified relative to those of temperate and tropical regions. The multiple exceptional ‘values’ of these ecosystems, in terms of features such as unique biodiversity, conservation, pristine nature and wilderness quality are widely recognized, as indicated by granting of status including UNESCO World Heritage, national park, nature reserve or protected area. The biodiversity of the terrestrial faunas of these regions is often dominated by invertebrate groups to a greater extent than is typical elsewhere. Many of these cold ecosystems are also, in effect, isolated island ecosystems, with recognized particular vulnerability to invasions by non‐native species (McGeoch et al., 2015). Here, we used the remote sub‐Antarctic Kerguelen and Crozet archipelagoes to explore the responses of major elements of their terrestrial invertebrate faunas to experimental exposure to predicted climate change. By working with short‐term ‘extreme warming events’, we unexpectedly identified a taxonomic component to the responses observed. A key element was that flies (Diptera) showed similar poor ability to survive warming conditions repeated on a daily basis, even when moderate, whether native or non‐native to the islands. In contrast, both native and non‐native beetles showed little or no evidence of deleterious consequences of increased exposure to short term warming. As dipterans are a major component of sub‐Antarctic, Arctic and other cold ecosystems (e.g. Chown & Convey, 2016; Coulson et al., 2014), such observations suggest that the increased occurrence of short‐term warming events could lead to large‐scale restructuring of key terrestrial ecosystem components, both in ecosystems protected from and those exposed to the additional impacts of biological invasions. With many of the likely impacted species playing a functional role in decomposition, we predict that warming may have major consequences for decomposition processes, with potentially serious consequences for trophic webs. The impacts of warming could be even more negative for dipterans if individual fertility is lost at thermal ranges lower than sub‐critical temperatures affecting survival rates.

## AUTHOR CONTRIBUTIONS

**David Renault**, **Hervé Colinet:** Conceptualization; methodology. **Aude Boutet**, **Camille Leclerc**, **David Renault**, **Hoel Hotte** and **Marc‐Antoine Colleu:** Data collection; curation. **Camille Leclerc** and **Steven L. Chown:** Formal analysis. **David Renault:** Funding acquisition. **Camille Leclerc**, **David Renault** and **Peter Convey:** Writing – original draft. **Camille Leclerc**, **David Renault**, **Hervé Colinet**, **Hoel Hotte**, **Peter Convey** and **Steven L. Chown:** Writing – review and editing.

## CONFLICTS OF INTEREST

The authors declare that they have no conflict of interest.

## INFORMED CONSENT

Informed consent was obtained from all individual participants included in the study.

## Supporting information


Supplementary Material S1

Supplementary Material S2

Supplementary Material S3

Supplementary Material S4

Supplementary Material S5

## Data Availability

The R scripts and data are available at: https://doi.org/10.5281/zenodo.6809341.
