# Supplementary material for: The rising threat of climate change for arthropods from Earth's cold regions: Taxonomic rather than native status drives species sensitivity
Source: Glob Chang Biol. 2022 Jul 22;28(20):5914–27. doi: 10.1111/gcb.16338 (PMC9544941; doi:10.1111/gcb.16338)
Supplement: Supplementary file 1 — Supplementary Material S1 [file GCB-28-5914-s005.docx]

**Supporting Information – The rising threat of climate change for arthropods from Earth’s cold regions: Taxonomic rather than native status drives species sensitivity**

**Table S1**. Details of the nine arthropod species whose thermal sensitivity to warming was assessed, including the years of collection and implementation of the survival assays. The numbers of individuals sampled to run the experiments is also indicated in parentheses. Condition ‘4-12’: from 4 to 12°C; Condition ‘4-20’: from 4 to 20°C; Condition ‘4-24’: from 4 to 24°C; Condition ’4-28’: from 4 to 28°C; NS: native species; NNS: non-native species.

|  | **Years of collection of the arthropods and implementation of the survival assays** | | | |
| --- | --- | --- | --- | --- |
| **Species** | **Condition C1** | **Condition C2** | **Condition C3** | **Condition C4** |
| **Diptera** | | | | |
| *Anatalanta crozetensis* (NS, Crozet) | 2016  (100 ind.) | 2016  (140 ind.) | 2015  (80 ind.) | 2015  (80 ind.) |
| *Anatalanta aptera* (NS, Crozet) | 2014 / 2016  (91 / 100 ind.) | 2014 / 2016  (91 / 140 ind.) | 2014 / 2015  (99 / 80 ind.) | 2014 / 2015  (102 / 80 ind.) |
| *Anatalanta aptera* (NS, Kerguelen) | 2014 / 2015  (101 / 100 ind.) | 2014 / 2015  (95 / 58 ind.) | 2014 / 2015  (98 / 100 ind.) | 2014 / 2015  (100 / 100 ind.) |
| *Amalopteryx maritima* (NS, Kerguelen) | 2015  (100 ind.) | 2015  (100 ind.) | 2015 / 2016  (101 / 100 ind.) | 2016  (100 ind.) |
| *Calycopteryx moseleyi* (NS, Kerguelen)* | 2014 / 2015  (99 / 80 ind.) | 2014 / 2015  (100 / 100 ind.) | 2014 / 2015  (100 / 100 ind.) | 2014 / 2015  (144 / 100 ind.) |
| *Calycopteryx moseleyi* (NS, Kerguelen)^Ф^ | 2014 / 2015  (100 / 100 ind.) | 2014 / 2015  (100 / 100 ind.) | 2014 / 2015  (100 / 100 ind.) | 2014 / 2015  (200 / 100 ind.) |
| *Fucellia tergina* (NNS, Kerguelen) | NA | NA | 2016  (101 ind.) | 2016  (100 ind.) |
| **Coleoptera** | | | | |
| *Amblystogenium pacificum* (NS, Crozet) | 2016  (100 ind.) | 2016  (140 ind.) | 2015  (80 ind.) | 2014 / 2015  (94 / 80 ind.) |
| *Bothrometopus brevis* (NS, Kerguelen) | NA | NA | 2016  (120 ind.) | 2016  (120 ind.) |
| *Merizodus soledadinus* (NNS, Kerguelen) | 2015  (100 ind.) | 2015  (100 ind.) | 2015  (100 ind.) | 2015  (100 ind.) |
| **Araneae** | | | | |
| *Myro kerguelenensis* (NS, Crozet) | 2014  (100 ind.) | 2014  (99 ind.) | 2014 / 2015  (99 / 70 ind.) | 2014 / 2015  (100 / 80 ind.) |

* Foreshore ecotype; ^Ф^ Kerguelen cabbage ecotype
