# Supplementary material for: The rising threat of climate change for arthropods from Earth's cold regions: Taxonomic rather than native status drives species sensitivity
Source: Glob Chang Biol. 2022 Jul 22;28(20):5914–27. doi: 10.1111/gcb.16338 (PMC9544941; doi:10.1111/gcb.16338)
Supplement: Supplementary file 3 — Supplementary Material S3 [file GCB-28-5914-s001.docx]

**Supporting Information – The rising threat of climate change for arthropods from Earth’s cold regions: Taxonomic rather than native status drives species sensitivity**


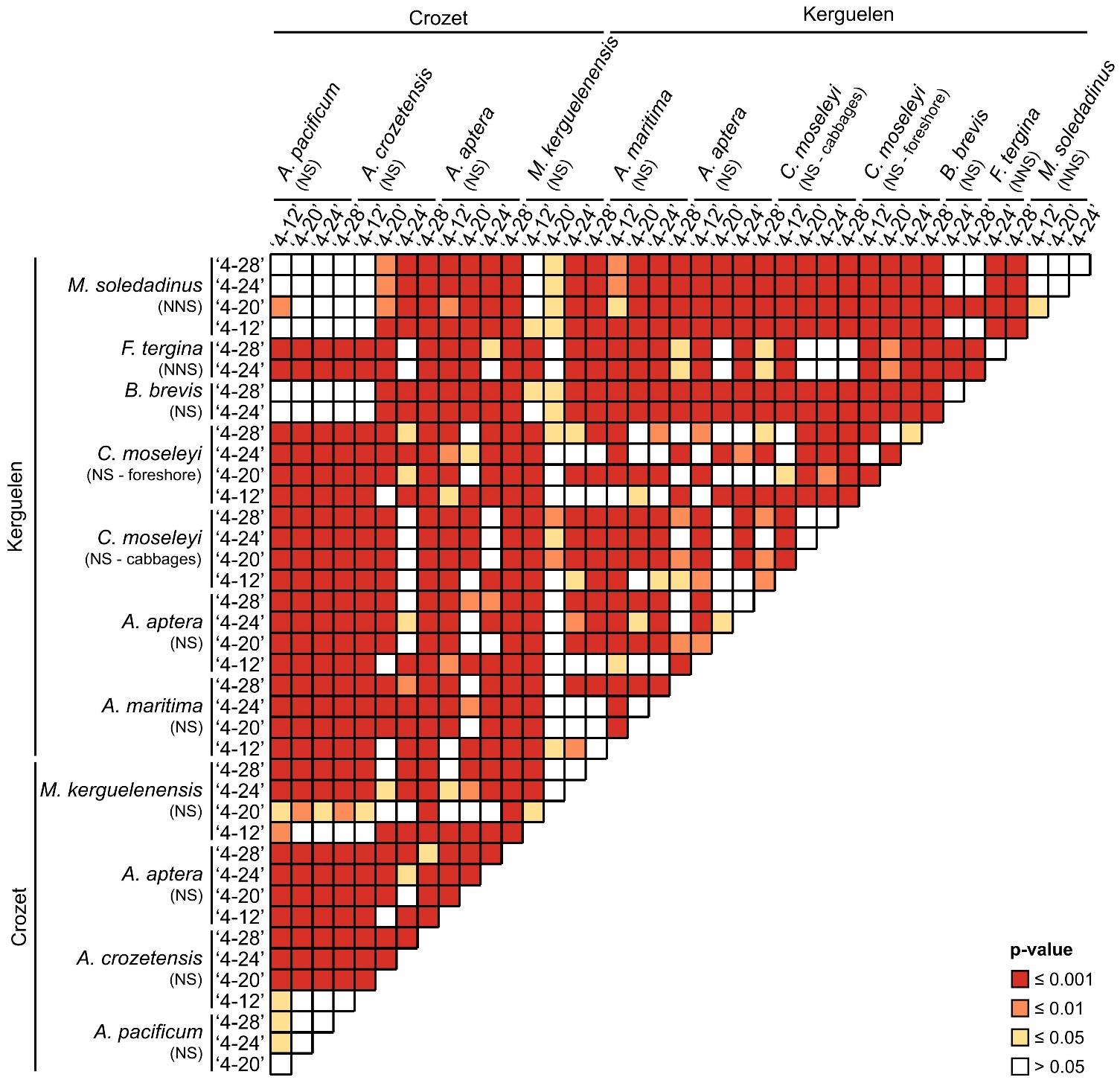


**Supplementary Material S3**. Pairwise comparison matrix. Multiple comparisons of survival curves among native and non-native arthropods from the Kerguelen and Crozet Islands under different experimentally-applied warming scenarios. Half heatmap is represented with color codes reporting statistical differences (range of values obtained for P): orange and red squares indicate significant differences among the compared pairs. The calculated pairwise comparisons were corrected for multiple testing using the Bonferroni correction.

Condition ‘4-12’: from 4 to 12°C; Condition ‘4-20’: from 4 to 20°C; Condition ‘4-24’: from 4 to 24°C; Condition ’4-28’: from 4 to 28°C; NS: native species; NNS: non-native species.
