# Supplementary material for: The rising threat of climate change for arthropods from Earth's cold regions: Taxonomic rather than native status drives species sensitivity
Source: Glob Chang Biol. 2022 Jul 22;28(20):5914–27. doi: 10.1111/gcb.16338 (PMC9544941; doi:10.1111/gcb.16338)
Supplement: Supplementary file 4 — Supplementary Material S4 [file GCB-28-5914-s002.docx]

**Supporting Information – The rising threat of climate change for arthropods from Earth’s cold regions: Taxonomic rather than native status drives species sensitivity**


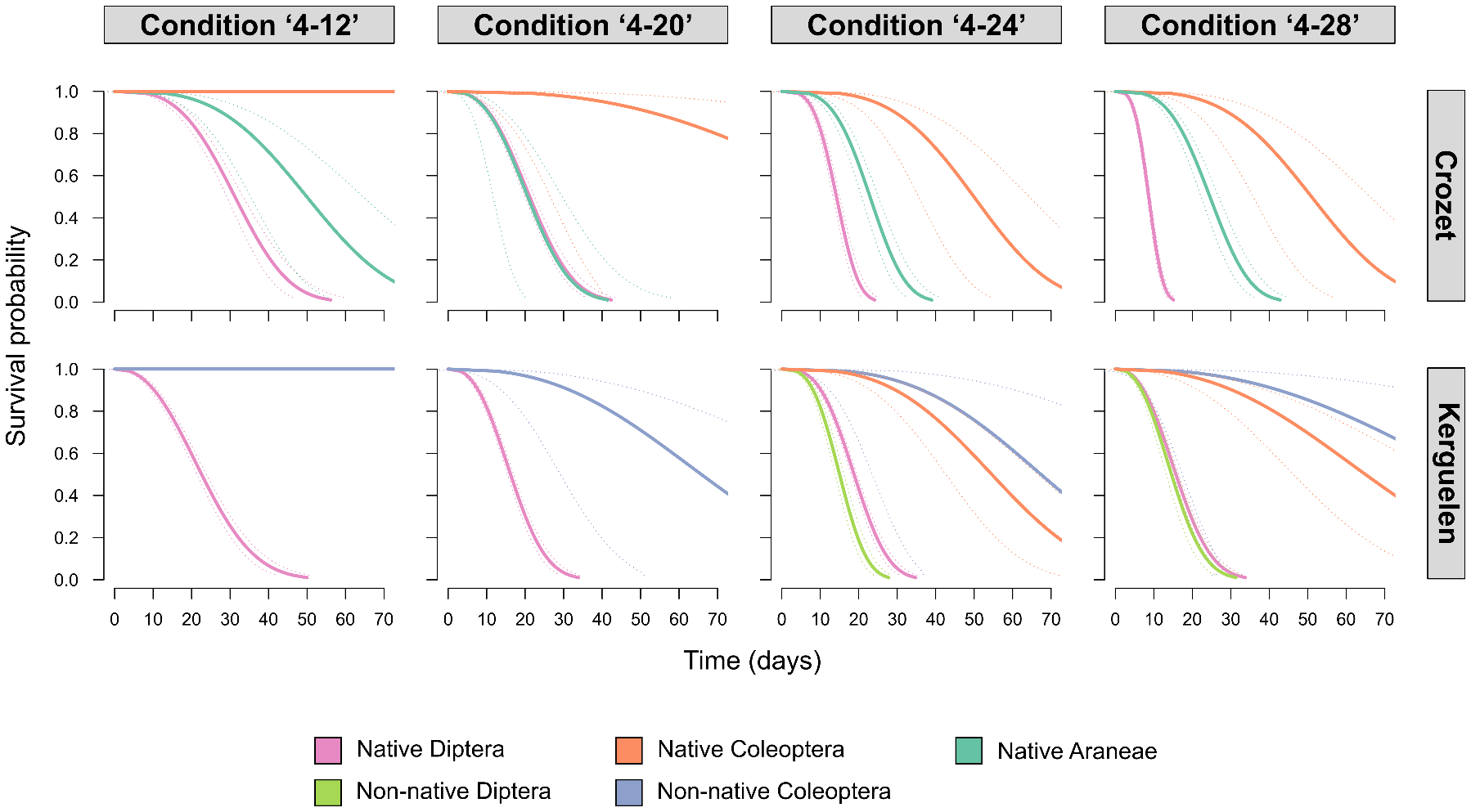


**Supplementary Material S4**. Fitted survival curves (Weibull distribution) of native and non-native arthropods, grouped according to taxonomic order, from Kerguelen and Crozet Islands under different experimentally-applied warming scenarios. Solid lines represent predicted values of survival probability and associated dotted lines represent 95% confidence intervals.

Condition ‘4-12’: from 4 to 12°C; Condition ‘4-20’: from 4 to 20°C; Condition ‘4-24’: from 4 to 24°C; Condition ‘4-28’: from 4 to 28°C.
