# Supplementary material for: The rising threat of climate change for arthropods from Earth's cold regions: Taxonomic rather than native status drives species sensitivity
Source: Glob Chang Biol. 2022 Jul 22;28(20):5914–27. doi: 10.1111/gcb.16338 (PMC9544941; doi:10.1111/gcb.16338)
Supplement: Supplementary file 5 — Supplementary Material S5 [file GCB-28-5914-s003.docx]

**Supporting Information – The rising threat of climate change for arthropods from Earth’s cold regions: Taxonomic rather than native status drives species sensitivity**

**
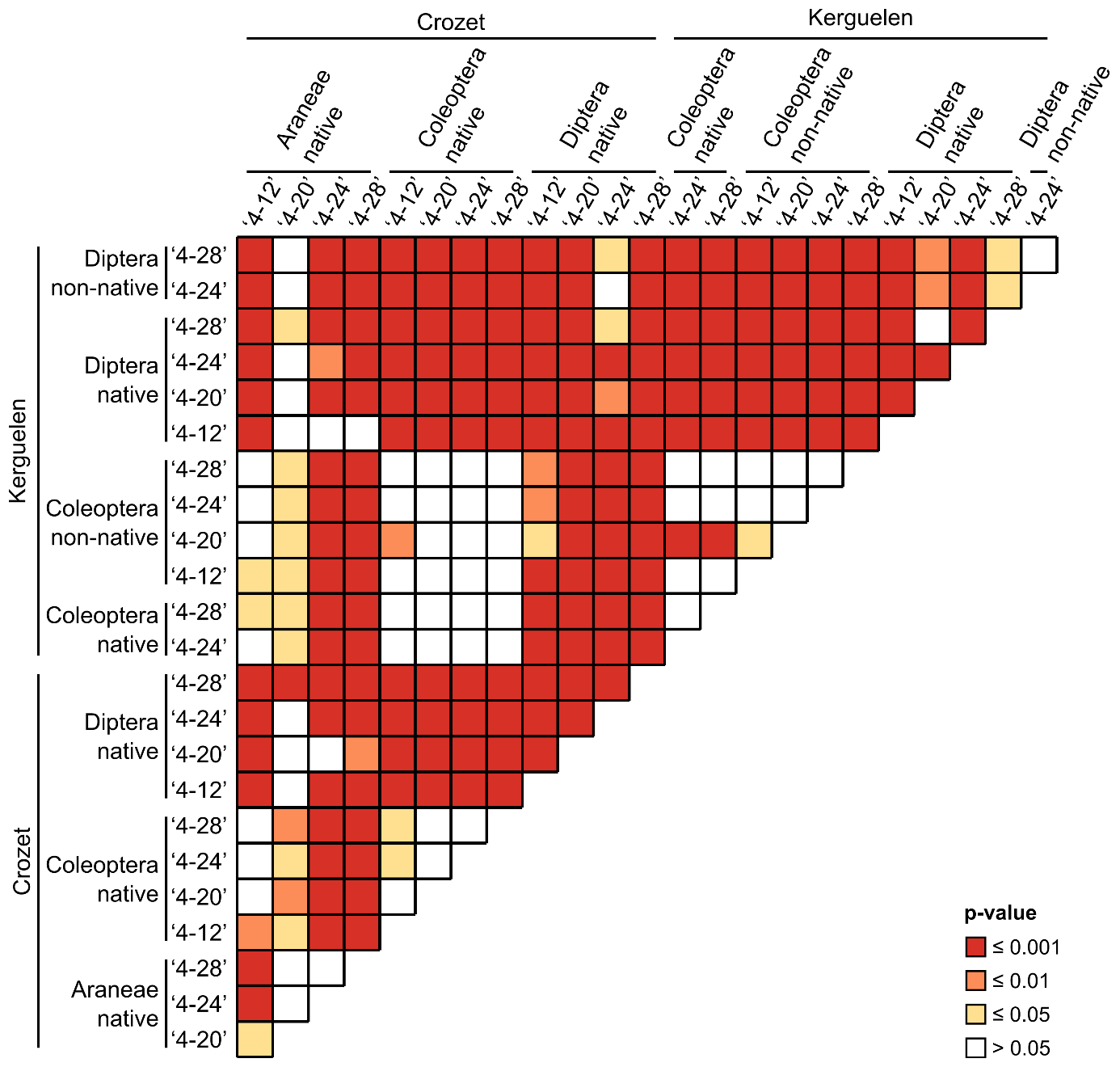
**

**Supplementary Material S5.** Pairwise p-value matrix from log-rank test. Survival curves of native and non-native arthropods, grouped according to taxonomic order, from Kerguelen and Crozet Islands under different experimentally-applied warming scenarios are compared. Under H_0_ the survival curves do not differ. The test is described in the Methods section. The colored squares on the half heatmap indicate statistical differences in survival curves between the two 'taxon – warming scenario' combinations while white squares indicate no significant difference. The calculated pairwise comparisons were corrected for multiple testing using the Bonferroni correction. Condition ‘4-12’: from 4 to 12°C; Condition ‘4-20’: from 4 to 20°C; Condition ‘4-24’: from 4 to 24°C; Condition ’4-28’: from 4 to 28°C.
